# Role of cholesterol in parasitic infections

**DOI:** 10.1186/1476-511X-4-10

**Published:** 2005-05-09

**Authors:** Devendra Bansal, Harinderpal Singh Bhatti, Rakesh Sehgal

**Affiliations:** 1 Department of Parasitology, Post Graduate Institute of Medical Education & Research, Chandigarh, India; 2 Central Research Institute, Kasauli, Himachal Pradesh, India

**Keywords:** Protozoa, Helminths, Pathogenesis, Lipids/Cholesterol

## Abstract

The requirement of cholesterol for internalization of eukaryotic pathogens like protozoa (*Leishmaniasis, Malaria *and *Toxoplasmosis*) and the exchange of cholesterol along with other metabolites during reproduction in *Schistosomes *(helminths) under variable circumstances are poorly understood. In patients infected with some other helminthes, alterations in the lipid profile have been observed. Also, the mechanisms involved in lipid changes especially in membrane proteins related to parasite infections remain uncertain. Present review of literature shows that parasites induce significant changes in lipid parameters, as has been shown in the in vitro study where substitution of serum by lipid/cholesterol in medium and in experimental models (in vivo). Thus changes in lipid profile occur in patients having active infections with most of the parasites. Membrane proteins are probably involved in such reactions. All parasites may be metabolising cholesterol, but the exact relationship with pathogenic mechanism is not clear. So far, studies suggest that there may be some factors or enzymes, which allow the parasite to breakup and consume lipid/cholesterol. Further studies are needed for better understanding of the mechanisms involved in vivo. The present review analysis the various studies till date and the role of cholesterol in pathogenesis of different parasitic infections.

## Introduction

Parasitic protozoa and helminthes are responsible for some of the most devastating and prevalent diseases of humans, threatening the lives of nearly one-third of the worldwide human population leading to more than 2 million deaths annually. Habitats of parasites are extremely varied and common parasites of man (protozoa, helminthes and arthropods) normally inhabit the intestine, blood, liver, lungs brain, muscles and lymphatic tissues [1]. Many species of parasites have complex life cycles involving developmental stages that live in soil or water, or use various kinds of intermediate hosts, including vertebrates and invertebrates and cold and warm-blooded animals. In such varied environments, parasites have become adapted to using/tolerating widely differing oxygen, carbon dioxide, hydrogen ion concentrations and temperatures [1]. Their nutritional requirements and their means of obtaining and utilizing the nutrients required for growth, motility and reproduction are also varied. The requirement of cholesterol for internalization of eukaryotic pathogens under such variable circumstances is poorly understood.

The present review highlights the role of lipids and their metabolical mechanisms in protozoan and helminthic infections.

Cholesterol is a major constituent of eukaryotic membranes and plays a crucial role in cellular membrane organization, dynamics, function and sorting. It is often found distributed non-randomly in domains in membranes [2]. Recent observations suggest that cholesterol exerts many of its actions by maintaining a specialized type of membrane domain, termed "lipid rafts" in a functional state. Lipid rafts are enriched in cholesterol and sphingolipids, and have been thought to act as platform through which signal transduction events are coordinated and pathogens gain entry to infect host cells [3].

Relationship of serum cholesterol levels in man infected with parasites has drawn the attention of various workers. Since it has been shown in-vitro studies that parasites like *Giardia *and *Entamoeba *can grown in lipid rich media in the absence of serum, it would be interesting to determinate the mechanism of lipid/cholesterol utilization. Recent studies have shown elevated levels of lipoproteins like high density lipoprotein (HDL), low density lipoprotein (LDL) and total cholesterol in patients suffering from parasitic infection (4). In human body cholesterol is synthesized in liver, which incidentally happens to be a major extraintestinal site of infection with *Entamoeba histolytica*. Keeping this in view, the following queries arise (i) is there any correlation between cases of amoebic liver abscess (ALA) with cholesterol synthesis in liver? (ii) in case of intestinal amoebiasis, what is the source of cholesterol? (iii) how do the amoebae utilize cholesterol in these two different areas (iv) what is the role of cholesterol in enhancing virulence and pathogenecity of *E. histolytica *(v) does cholesterol help in cyst formation?

*Entamoeba*, *Giardia *and *trichomonads *form what can be usually termed as 'mucosal parasites' which lack mitochondria, well developed golgi complexes, and other organelles typical of higher eukaryotes [1]. These parasites have developed unique metabolic pathways that allow them to survive and multiply by scavenging nutrients from the host, but are unable to synthesize the majority of their own lipids and cholesterol de novo [5]. Therefore, an understanding how they transport and utilize exogenous lipids for metabolic purposes is extremely important? There is evidence suggesting that these parasites can take up the lipids and cholesterol they need from lipoprotein particles present in the host gut and tissue in vivo and from the growth medium in vitro. Exogenous phospholipids have been shown to undergo fatty acid remodeling [by deacylation/reacylation reactions], which allow these protozoa to alter lipids, bypassing the synthesis of entirely new phospholipid molecules [5].

The endocytic process is essential for uptake of nutrients and other molecules by protozoan parasites. Membrane dynamics in endocytosis has been investigated in parasites such as *Leishmania *and *Trypanosoma*. The receptor-mediated endocytosis of LDL by *Schistosoma mansoni *has been reported [6]. A putative LDL-receptor has been identified on the tegument and gut lining from *Schistosoma japonicum *adult worms [7] and schistosomula of *S. mansoni *[8]. Recently, Coopens et al [9] demonstrated that the intracellular parasite, *Toxoplasma gondii*, acquires host cholesterol that is endocytosed by the LDL pathway, a process that is specifically increased in infected cells. Interference with LDL endocytosis or cholesterol translocation reduced the intracellular survival of *T. gondii *[9]. In a classic experiment, Aley et al [10] demonstrated the phenomenon of endocytosis through non-acidified vesicles in *E. histolytica*. They showed that these non-acidified vesicles (distinct from intracellular acidified vesicles) were derived from plasma membranes and played important roles in endocytosing nutrients and other biological molecules into cells.

### Cholesterol – biosynthesis and metabolism

Cholesterol is an amphipathic lipid and as such is an essential structural component of all cell membranes and of the outer layer of plasma lipoproteins. It is present in tissues and in plasma lipoprotein either as free cholesterol or, combined with a long-chain fatty acid, as cholesteryl ester. It is synthesized in many tissues from acetyl-CoA and is ultimately eliminated from the body in the bile as cholesterol or bile salts [11]. Lipoprotein transports free cholesterol in the circulation, where it readily equilibrates cholesterol in other lipoproteins and in membranes. Cholesteryl ester is a stored form of cholesterol found in most tissues. It is transported as cargo in the hydrophobic core of lipoproteins [12]. So, the fact that cholesterol in a hydrophobic molecule, which resides in lipoproteins and cell membranes, raises two questions: (i) how does the cell sense the level of cholesterol? (ii) how in this cholesterol specific signaling transmitted to the nucleus for the regulation of various genes?

Recent studies directed to resolve these questions, led to the discovery of a novel cell surface cholesterol-sensor designated as receptor-Ck which was not only shown to be ubiquitously present in various human organs but also [through its signaling pathway] regulated various genes involved in cholesterol homeostasis [HMG CoA synthase; HMG CoA reductase; apo 'B'- specific LDL – receptor] cell growth [cyclin 'D'; C – fos; C-myc; p27 etc]; cell death [Bc1-2] through a 47 KDa transcription factor [derived from the cleavage of 125 KDa SREBP] having affinity for genomic sterol regulatory element [SRE] sequence as well as through other transcription factors [13-17].

## Anaerobic parasites

### Entamoeba

*Entamoeba histolytica *parasitizes the gastro-intestinal tract of humans and is a major cause of morbidity and mortality in tropical and subtropical countries [18]. Although several successive life cycle stages of *E. histolytica *have been documented, briefly, it can be classified into two main morphologic forms, i.e. trophozoites and cysts. In *E. histolytica*, excystation occur in the small intestine, where four amoebae are released from the mature quadri nucleate cyst. Trophozoites dwell in the colon, where they multiply and encyst typically producing four nucleated cyst [19]. Cholesterol has been reported to be a growth – promoting factor of *E. histolytica *[20]. The virulence of a virulent strain could be revived in vitro by adding cholesterol to medium or in vivo by feeding cholesterol to experimental animals or the host [21-23]. Cholesterol is thought to act as an irritant to mucus membrane and thereby helps the amoebae to establish and colonize at the injured site and this enhances the virulence [24]. In-vitro study has shown that substitution of lipid/cholesterol instead of serum, supported vigorous growth of *E. histolytica *in axenic culture [25].

Recently, Laughlin et al [26] have shown that the disruption of cholesterol rich raft-like membrane domains in *Entamoeba *with the cholesterol-binding agent filipin and methyl-β-cyclodextrin inhibit several important virulence functions, fluid phase pinocytosis and adhesion to host cell monolayers. However, disruption of raft-like domains did not inhibit constitutive secretion of cysteine proteases, another important virulent function of *Entamoeba*. Cysteine proteinases in *Entamoeba *are likely to be associated with tissue invasion and pathogenesis [27]. Andra et al [28], revealed that the lipid composition of amoebic membranes prevents binding of the cytolytic molecules and that both the phospholipid ingredients and the high content of cholesterol contributes to the protection of the toxin-producing cell.

More recently the authors have assessed the impact of lipid parameter in patients infected with *E. histolytica *and *E. dispar*. The result showed significant lower levels of lipid profile [total cholesterol, HDL & LDL] in *E. histolytica *and *E. dispar *cyst passers and ALA patients as compared to healthy controls. Also, cyst passers had lower levels of cholesterol than ALA cases [29].

### Giardia

Giardiasis is the most common waterborne disease in human, which is caused by an enteric flagellated protozoan *Giardia lamblia*. *Giardia *is widespread with children being the most vulnerable [30]. *Giardia *exists in two morphologic forms: trophozoite and cysts. In *G. lamblia*, excystation is accomplished in two steps first by limiting the acidic conditions present in the stomach and secondly by the protease-rich and slightly alkaline small intestine. Encystation, in turn, can be induced in vitro by starving the trophozoites of cholesterol, either by using lipoprotein-deficient serum or augmenting the bile concentration in the culture medium [31,32]. Membrane biogenesis in *Giardia *requires cholesterol [33,34]. Because *Giardia *is unable to synthesize cholesterol [33] it obtains the same from upper small intestine, which is rich in biliary and dietary cholesterol [35,36]. Effects of bile salts on encystations are directly related to the uptake of cholesterol by the trophozoites [37].

In vitro, it was observed that replacing bovine serum with a lipoprotein cholesterol (LPC) solution and bovine serum albumin in pre-encystation and excystation media, stimulated *G.1amblia *encystations and encystation specific secretory vesicles (ESV) formation [38]. It is established that cholesterol-dependent down-regulation of encystations-specific CWP-1 gene expression has contributed to the inhibition of *Giardia *encystation process [31,37]. Previously, we have reported that receptor Ck dependent signaling is responsible for the regulation of Giardia encystations process by cholesterol [39]. Recently we have observed that patients infected with *G. lamblia *showed lower levels of lipid parameters (total cholesterol, HDLc and LDLc) as compared to control healthy group [29].

### Trichomonas

*Trichomonas vaginalis *is a sexually transmitted protozoan parasite, adheres to the vaginal epithelium, causing vaginitis and other complications in women [40]. It is an anaerobic protozoan flagellate, which lacks mitochondria and peroxisomes, but has a specialized double-membrane-bounded organelle called the hydrogenosome, which is involved in metabolic processes that extend glycolysis [41]. In vitro study has shown that when serum was replaced by bovine serum albumin and cholesterol, it resulted in good growth [42].

## Apicomplexan parasites

### Plasmodium

*Plasmodium falciparum *is the most pathogenic species causing human malaria. Erythrocytes become infected following attachment and invasion by merozoite. Various phases of parasite development can be observed during the 48 h – erythrocytic cycle i.e. rings, trophozoites and schizonts. In vitro the parasite grows in 5–10% human serum in an atmosphere of low oxygen [43]. Very little is known about mechanism involved in lipid changes related to malaria. Hypercholesterolemia and hypertriglyceridemia was observed in both uncomplicated and complicated malaria [44-46], whereas Kittl et al [47], have shown no correlation between severity of malaria attacks and extent of HDL – cholesterol decrease. Human serum HDL is necessary for *P. falciparum *in *in vitro *culture. However, it has been reported that HDL can be toxic for the parasite at high concentrations [48].

Recently, Imrie et al [49], have also reported that in the absence of serum, HDL in low concentration (0.75 mg/ml) supported growth of *P. falciparum *in vitro, whereas at high concentration (3 mg/ml), it was toxic to the parasite. Recent findings, however, would suggest that the *plasmodium *genome contains genes encoding enzymes of phospholipids metabolism, allowing de novo synthesis of phosphatidyl choline via the knneddy pathway and necessitating only the uptake of the small choline molecule [50]. In addition, the genome of *P. falciparum *contains genes similar to those for type II fatty acid synthesis pathway. The protein products of these genes are located within the apicoplast and allow for the production of fatty acids, some of which are unique to the parasite [50]. Thus the parasite may be able to meet many of its lipid requirements from its own biosynthetic pathways, although some extracellular lipids are necessary for in vitro growth. It has also been seen that plasma membrane cholesterol plays a role in the pathogenesis of immune evasion and clinical manifestations of falciparum malaria [51].

There are few studies which suggest that there are changes in lipid plasma or serum levels in-vivo after infection. But, no significant changes were seen in the plasma cholesterol during and after infection of malaria [52]. However, low levels of the cholesterol in patients infected with malaria as compared to normal healthy controls have been reported [53]. In another study changes in plasma lipoprotein was seen in acute malaria resulting decreased levels of HDL and LDL and moderately increased triglycerides [54]. In malaria endemic areas, when plasma levels of cholesterol, triglcerides, HDLc and LDLc were analysed in children infected with *P. falciparum*, investigators have found significantly low levels of lipid profile [55]. Brotons et al [56] reviewed that population studies on common lipid parameters and observed that cholesterol values are lower in Africa, where, malaria is endemic, than in many other parts of the world.

### Toxoplasma

*Toxoplasma gondii*, an apicomplexan protozoan parasite, is an important pathogen of humans and animals. It is widely distributed with a very high prevalence in many regions, and can cause serious infections in immuno-compromised patients (particularly AIDS patients) and in the developing fetus [57]. Host cell cholesterol is implicated in the entry and replication of an increasing number of intracellular microbial pathogens. However, recently new mechanism has been described by which host cholesterol specifically controls entry of an intracellular pathogen. Briefly, the parasitophorus vacuole membrane (PVM) surrounding *T. gondii *contains cholesterol. At the time of cell entry host plasma membrane cholesterol is incorporated into the forming PVM during invasion, through a caveolae independent mechanism. Depleting host cell plasma membrane cholesterol blocks parasite internalization by reducing the release of rhoptry proteins that are necessary for invasion [58].

In another study, Coppens et al [9] had shown that *T. gondii *exploits host low density lipoprotein receptor-mediated endocytosis for cholesterol acquisition. Whereas, acyl-CoA: cholesterol acyl transferase (ACAT) and cholesterol esters play a crucial role in the optimal replication of *T. gondii *[59]. These studies indicate the cholesterol does have a role in the pathogenesis of *Toxoplasmosis*.

### Cryptosporidium

Heterogeneous distribution of membrane cholesterol at the attachment site of *Cryptosporidium muris *to host cells has already been investigated. Although many filipin-cholesterol complexes were observed on the plasma membrane of host cells and parasites, a line showing no complexes was evident at the above two membrane junctures. These observations indicate that parasitic infection of *C. muris *altered the organization of membrane cholesterol [60]. The exact role of cholesterol in pathogenesis/virulence of parasite needs to be determined.

## Kinetoplastid parasites

### Leishmania

*Leishmania *is an obligate intracellular parasite that infects macrophages of the vertebrate host, resulting in visceral, cutaneous and mucocutaneous leishmaniasis in humans. Recently Pucadyil [61] reported that plasma membrane cholesterol is required for efficient attachment and internalization of the parasite in macrophages, leading to *Leishmania donovani *infection. Rodrigues et al [62] have shown that when amastigotes and promastigotes forms of *L. amzonensis *are incubated with 22,26-azasterol, which is a delta (24(25))-sterol methyltransferase (SMT) inhibitor, it results in arrest of growth. They also observed that alteration occur in the lipid composition of the parasite membrane, resulting in loss of viability. The study suggests the use of 24-SMT inhibitor could be used as selective antileishmanial agent. Dietz et al [63] have reported successful treatment of Brazilian kala-azar using lipid encapsulated amphotericin B. Thus, a level of cholesterol in the patients infected with leishmaniasis has to be determined.

### Trypanosoma

African trypanosomes are lipid auxotrophs that live in the bloodstream of their human and animal hosts and are unable to synthesize cholesterol but appear to bind and take up plasma low-density lipoproteins (LDL) from their host [64]. Whether cholesterol homeostasis of this unicellular parasite also requires interactions with host high-density lipoprotein (HDL) particles is unknown. Trypanosomes require lipoproteins to multiply under axenic culture conditions [65]. Recently, Green et al [66] reported that HDL, LDL, and trypanosome lytic factor (TLF1) were bound and taken up by a lipoprotein scavenger receptor, which may constitute the parasite's major pathway mediating the uptake of essential lipids. Frequent turnover of variable surface glycoproteins is a well established method of immune evasion by *Trypanosomes*. Does it involve the role of HDL, LDL, and TLF1 in getting eliminated by attaching to the surface receptors of Trypanosomes?

## Helminthes

### Schistosoma

It has been shown that *Schistosome *infection could be counteracting the effects of an atherogenic diet by modulating host lipid metabolism and inducing a reduction in blood total cholesterol concentration [67]. Little information is available on the lipid changes caused by *S. mansoni *reinfection. Popiel et al [68], studied the metabolism of cholesterol uptake by paired and unpaired worms of *S. mansoni *during pairing, the results showed labeled worms lost upto 65% of their cholesterol. This suggests that normal cholesterol transfer in worm pairs is bi-directional and that it is facilitated by physical contact between juxtaposed membranes. Cholesterol exchange in schistosome worm pairs may be partly or wholly consequences of normal tegumental turnover of the molecule.

In another study Silveira et al [69] investigated the transfer of cholesterol and its metabolites between adult male and female worms of *S. mansoni*. They found that the adult male and female worms of *S. mansoni *are able to incorporate cholesterol and convert it into several metabolites. On the other hand, *Schistosomula *cannot convert the incorporated cholesterol. However, a significant reduction in levels of serum lipid profile was observed in mice infected with *S. mansoni *[70]. These changes might be attributed to several metabolites released by *S. mansoni*, which affect the host hepatic tissue resulting in decreased synthesis of these parameters and their release into the circulation. The ability of *Schistosomula *to convert cholesterol into its metabolites, shows that it is a property acquired, used by adults only and is specifically active during pairing. What is the exact role of cholesterol during pairing needs to be investigated. What happens to cholesterol levels in serum of patients also needs to be studied.

### Intestinal worms

In-vivo study has shown the decreased serum lipid levels in the Shipibo population (Peru), showed a significant inverse correlation between worm egg excretion and HDL levels in hookworm, *Strongyloides *and *Trichuris *infected patients but not in *Ascaris *infected cases [71]. The mechanisms underlying the observed association between intestinal worm load and HDL reduction are not completely understood and may include reduced HDL synthesis in the gut wall due to inflammatory toxic irritation. Hence, cholesterol may have a role in pathogenesis by helping the larvae to survive in the host tissue.

### Ascaris

During larval ascariasis the metabolism of lipids is significantly disturbed. Decreased levels of total cholesterol, HDL cholesterol and triglycerides were observed in guinea pigs. The changes are due to the break in liver function and, presumably, changes in hormone secretion, which are provoked by the presence of the parasite [72]. It has also been seen that cholesterol enhanced larval survival and the yield and growth of L4 of *A. summ *larvae when added to RPMI-1640 culture medium [73].

### Ancylostoma

In vitro *A. duodenalis *grown axenically in medium supplemented with cholesterol gives good growth [74]. Phospholipid/cholesterol ratio in liver plasma membrane of the infected golden hamsters with *A. ceylanicum *group was significantly reduced, which suggest that both the structural and functional organization of membrane may be a biochemical basis of the hepatotoxic effects [75].

### Filaria

Decreased levels of lipid contents were observed in liver of *Mastomys natalensis *during the development of *B. malayi *infection [76]. The lipid peroxide formation was enhanced in liver during the development of filarial infection. The studies in human beings are lacking.

### Hymenolepis

Balb/c mice infected with *Hymenolepis microstoma *significantly affected the lipid metabolism [77]. The changes were in relation to nutritional interactions between host and parasite and the possible effect on host hormone levels. Johnson et al [78] studied on the mechanisms of cholesterol uptake by the rat tapeworm *H. diminuta*. The results support the hypothesis that the tapeworm absorbs cholesterol by a specific carrier-mediated process. Cholesterol uptake is reduced when the capacity of the micellar phase of the medium is increased, suggesting that uptake involves the intermediate partitioning of sterol from micelles into the aqueous phase of the medium [79].

## Conclusion

The mechanisms involved in lipid changes related to parasite infections remain uncertain. Cholesterol starvation initiates encystations. This indicates that cholesterol has a role in pathogenesis as it helps the parasite to remain in trophozoite stage. However, many workers have investigated that medium containing lipoproteins supported the growth of the parasites in vitro. It is possible that all these parasites use LDL-like receptors to endocytose various lipoprotein particles. However, typical receptor binding affinity, number of LDL-receptors exposed on cell surfaces, receptor internalizations etc. in mucous-dwelling parasites are not known. Few population based studies showed altered lipid profiles in patients infected with malaria compared to asymptomatic control group. So, to evaluate the impact of malaria on lipid parameters at the population levels in different age groups, studies are needed to explore the relevance of this finding in different patterns of hyperendemic regions. The association of parasites and the contents of their membranes before penetration/invasion and realignments seen after attachment need to be studied. Also, the role of PVM and their contents in protection of parasites within PVM also need to be studied.

So far, the studies suggest that there may be some factors or enzymes, which allow the parasite to breakup and consume lipid/cholesterol. Further studies are needed for better understanding of the mechanisms involved in vivo.

## Future strategies

The evidence suggests that parasites are able to remodel/metabolize host lipids for their growth and to generate phospholipids membrane. So, the identification and characterization of more enzymes involved in these pathways will be more important for the proper understanding of these steps. Recent progress in molecular biology and parasite genome projects will assist researchers in the near future to identify the genes and enzymes of lipid metabolic pathways.
